# Variation Pattern of the Compressive Strength of Concrete under Combined Heat and Moisture Conditions

**DOI:** 10.3390/ma16041548

**Published:** 2023-02-13

**Authors:** Ping Li, Ji Liu, Shiwei Duan, Ruiyuan Huang

**Affiliations:** 1 School of Mechanical Engineering, Anhui University of Technology, Maanshan 243032, China; 2 College of Civil Engineering, Fuzhou University, Fuzhou 350116, China

**Keywords:** concrete compressive strength, temperature softening effect, water softening effect, heat–moisture coupling factor

## Abstract

The compressive strength of concrete is not the same in high temperature humid environments and normal temperature dry environments. In this study, quasi-static uniaxial compression experiments of concrete with different temperatures and water contents were carried out to investigate the variation pattern of the compressive strength of concrete under combined heat and moisture conditions. The results showed that the temperature softening effect and water softening effect of the compressive strength of concrete were coupled with each other. The compressive strength exhibited a variation trend from increase to decrease with the increase in both temperature and water content, and the relations among the heat–moisture coupling factor, temperature, and relative saturation ratio were obtained. The water absorption of concrete after immersion had a more significant effect on the compressive strength than the free water content stored inside the specimen before immersion. The “pseudo-temperature strengthening effect” distinguished the thermodynamic response of immersed concrete from that of dry concrete, and the functional relationships among the heat–moisture coupling factor, temperature, and relative water absorption ratio were established. The evolutionary mechanism of the competition between the microcrack expansion and healing of concrete under combined heat and moisture conditions was revealed.

## 1. Introduction

Concrete structures, with their advantages of better corrosion resistance, impact resistance, and high temperature resistance, have a wide range of applications in national defense constructions and civil engineering. Studies [1,2,3,4,5] have shown that when concrete works under extreme environmental conditions such as high temperature or fire, the water content inside the material has a non-negligible effect on the performance of concrete materials. This is especially relevant in the nuclear power industry, where concrete serves as the main sealing material of the pressure containment vessel of the nuclear reactor, and thus its water content is an important factor in reactor containment design. To provide adequate radiation protection, the concrete containment vessel is supposed to have the highest possible moisture content. In addition, when heated, the moisture inside the concrete helps to absorb the latent heat of evaporation, thereby retarding the inward flow of heat and providing high temperature protection. In accidents such as leakage of hot material inside the nuclear reactor vessel or cooling system failure, the temperature of concrete is higher than the allowable temperature under normal operating conditions (100 °C). Therefore, it is very important to understand how temperature and moisture influence the compressive strength of concrete materials. In recent years, with the progress in technology and development of the economy, experimental studies and numerical simulations of the thermodynamic properties of concrete have been conducted. Many scholars have researched the material properties of concrete under conditions of strength, heat, and moisture and have achieved valuable results. The quasi-static compressive strength of concrete at normal temperature decays with the increase in water content, which is due to the fact that the wedging action of water at the tip of the crack during the hydration process exacerbates the crack expansion, thereby reducing the strength of concrete [6,7,8]. From the perspective of the molecular structure, the dissolution of the intercrystalline bonds in the lattice of the concrete structure after water immersion causes a reduction in van der Waals forces, which also leads to a reduction in concrete strength. In addition, it is generally accepted that the reduction in concrete strength after water immersion is reversible, and when the material is dried, the concrete regains its original strength almost completely [9]. Research on the mechanical properties of concrete at high temperatures can be traced back to a century ago, and it has shown [10,11,12,13,14,15,16,17,18,19,20,21,22,23,24,25,26,27,28,29] that the strength of concrete materials has a significant temperature weakening effect at high temperatures. Ping Li [30] obtains the variation pattern of the temperature softening effect factor of the uniaxial static and dynamic compressive strength of concrete materials. This is achieved by summarizing the results of static and dynamic experiments of concrete at high temperatures reported by dozens of scholars in recent years.

**Figure 1:**
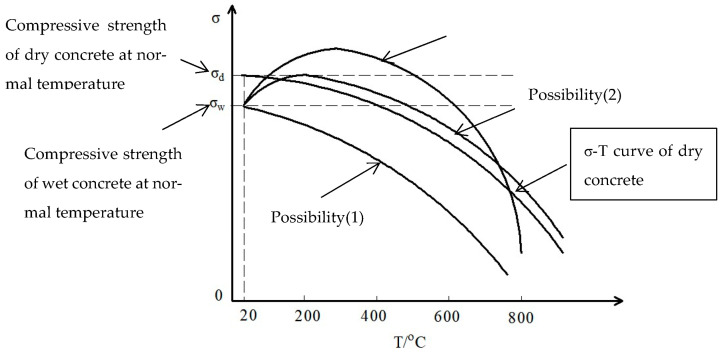
The schematic diagram of the possible relationship between compressive strength and temperature.

Compared to the research on the water softening effect of wet concrete at normal temperature and the temperature softening effect of dry concrete at high temperature, scholars are less unified in their views on the pattern of the effect of temperature and water on the compressive strength of concrete materials under combined heat–moisture conditions. Rather, there are uncertainties and controversies on some key issues. For example, some scholars believe that the main reason for the damage of concrete materials under high temperature is that the pore pressure inside the material increases due to the obstruction of water vapor diffusion under high temperature, thus leading to the phenomenon of high temperature bursting of concrete materials [31,32]. However, some scholars have different opinions on this view. Mindeguia [33] found that the pore pressure inside the immersed concrete at high temperature, measured through the pore pressure test device, is not strong and even lower than that of dry concrete under combined heat and moisture conditions. This proves that the damage of water-bearing concrete at high temperatures is not entirely the result of the action of pore pressure. Although the compression strength of concrete decreases under the action of temperature or water alone, the changing pattern of the compressive strength remains to be further determined in the process of the gradual loss of water inside the concrete with the increase in temperature under the combined heat and moisture conditions. The experimental results under combined heat and moisture conditions show the following possibilities:

(1) The effects of temperature and water on the strength of wet concrete are independent. The compressive strength of concrete is the superimposed result of the independent effects of the temperature softening effect and water softening effect. Due to the secondary hydration during water immersion, a large number of cracks occur within the concrete, which does not heal or shrink with the phase change and loss of water at high temperature. This means that the damage formed in the process of hydration is irreversible at high temperature. The compressive strength of concrete after immersion monotonically decreases with the increase in temperature.
(2) When wet concrete is exposed to high temperatures, the water is evaporated and the water content decreases. With the loss of free water, the splitting effect of water at the crack tip disappears, and the hydration cracks formed in the immersion process are completely healed, and the damage is reversible. The phase transition of water does not affect the healing of cracks caused by the loss of liquid water. The compressive strength of wet concrete gradually increases with the increase in temperature until the water absorbed during the immersion process is completely lost and the compressive strength reaches the peak, which is equal to the compressive strength of dry concrete at normal temperature. Then, it shows the same decay pattern as that of the compressive strength of dry concrete at high temperature. The absorbed water delays but does not affect the temperature softening effect on the compressive strength. The effect of water on the high temperature strength of concrete materials is fully reversible such as that of the compressive strength at normal temperature, that is, the phase transition of water has no effect on the damage of concrete materials and the water content has no effect on the peak strength and the attenuation pattern of post-peak strength.
(3) The effects of temperature and water on the strength of wet concrete are coupled. Under the action of high temperature, wet concrete gradually becomes dry concrete due to the evaporation of internal moisture. The compressive strength of concrete first increases to a certain peak strength and then decreases with the rise of temperature. Both the peak strength and the attenuation pattern of post-peak strength are related to the water content. The peak strength and attenuation pattern of post-peak strength are not the same as those of dry concrete under the same temperature conditions. The phase transition of water at high temperature has an irreversible effect on the evolution of cracks in concrete materials, at least on part of the crack evolution. The temperature and water effects on the compressive strength are interactive. Under the above three possibilities, the general relationship between the compressive strength and temperature is sketched as shown below (Figure 1):

In response to the above controversies and uncertainties and to accurately grasp the joint effect of temperature and moisture on the compressive strength of concrete materials under combined heat and moisture conditions, thus determining the variation pattern of compressive strength, this study carried out quasi-static uniaxial compression experiments of concrete under different temperatures (normal temperature, 200 °C, 400 °C, and 520 °C) and different water contents (stoved in oven, air drying at normal temperature, immersed in water for approximately 2 h, 6 h, 60 h, and fully saturated) and obtained the effects of temperature and water on the compressive strength of the concrete material. The different effects on the compressive strength under high temperature between the free water stored in the first hydration process of concrete and the water absorbed in the second hydration process of concrete were observed. The special phenomenon of “pseudo-temperature strengthening” was discovered in the concrete specimens after immersion. The heat–moisture coupling pattern of the compressive strength of concrete was determined, and the meso-evolution mechanism of microcracks inside the material under combined heat and moisture conditions was revealed.

## 2. Preparation and Testing of Specimens with Different Water Contents

The raw material for the specimens consisted of coarse aggregate (crushed stone with a maximum particle size of 8 mm), cement with a strength class of 42.5 MPa, medium sand with a fineness modulus of 2.3, and a small amount of water reducing agent. The mix proportions of the specimens are shown in Table 1. The specimens are cylinders with the size of ϕ50 mm × 100 mm. Meanwhile, in order to understand the influence of the size effect of specimens on water absorption, a group of cylindrical specimens with the size of 70 mm × 35 mm is added as comparison specimens.

In this experiment, the curing of concrete method adopted was in accordance with clause 5.2.4 of the “Standard of Test Methods for Mechanical Properties of Ordinary Concrete, GB/T50081” currently practiced in China. The standard curing environment of concrete specimens is: within 28 days, the temperature is maintained at 20 ± 2 °C, the relative ambient humidity is >95%, the specimens should not be stacked, the interval between each other is required to be 10–20 mm, and the surface should be kept wet, but not directly wet with water, but by using atomization humidification treatment. After the specimens were fabricated and cured in the standard way for 28 days, they were placed in a dry indoor environment at normal temperature for at least three months. The specimens under this condition were considered as air drying concrete specimens, whose mass was recorded as M_0_. The air-drying specimens were heated in the oven at 105 °C and were weighed and recorded several times during the heating process until the mass no longer changed. Then, they were considered completely stoved specimens, the mass of which was measured as M_d_. The mass of free water and the water content in the air-drying specimen were measured as δM (δM = M_0_ − M_d_) and δM/M_0_, respectively. The experiments showed that the water content stored in the air-drying specimen placed in normal temperature and dry environment for three months was approximately 1.87%.

The production process of the immersed concrete specimen included two hydration actions. The first hydration action occurred during the process of production and curing of the initial concrete. The initial concrete specimen was made in accordance with the predetermined mix ratio and cured for 28 days in the standard way. The initial specimens placed in a normal temperature dry environment for more than three months were referred to as air drying specimen. Then, the air-drying specimens were immersed in water to make immersed concrete specimens with different water contents. The second hydration occurred in the process of immersing the air-drying concrete specimen. The water content in the concrete specimen maintained in a dry room at normal temperature before immersion was called the initial water content, and the water content of the specimen after immersion increased with the increase in immersion time until saturation, when the mass of the specimen no longer increased with the increase in immersion time.

The air-drying specimen was immersed in water (the second hydration occurred inside the material during the immersion process). Different water contents inside the specimen were achieved by controlling the immersion time. The change in the mass of the specimen under different periods of immersion during the immersion process was measured, taking the mass of the air-drying specimen M_0_ as the reference. The water absorption mass ΔM of the specimen was obtained (ΔM = M − M_0_, where M is the mass of the concrete specimen after immersion for a predetermined time). The water absorption mass, ΔM, does not include the free water mass, δM, stored inside the air-drying specimen, but the water absorption mass of the specimen during the immersion process for a predetermined time. The water absorption ratio, S, is the water absorption mass ΔM/the air-drying specimen mass M_0_, i.e., S = ΔM/M_0_. The water content of the immersed concrete is: ΔM + δM, and the water content ratio, W%, is the mass of water in the concrete specimen/the mass of the concrete completely stoved in the oven, i.e., W% = (ΔM + δM)/M_d_.

The real time water absorption of the specimen is not only related to the immersion time, but is also affected by the time that the specimen is placed in the dry environment before immersing and the size, shape, and mix proportions of the specimen. In this experiment, a cylindrical specimen (specimen size: ϕ50 mm × 100 mm) placed in a dry environment for 3 months after standard curing and two sets of cylindrical specimens of different sizes (ϕ70 mm × 35 mm and ϕ50 mm × 100 mm) placed for 9 months, all of which were obtained by coring from the same concrete cube embryo, were tested for the variation law of water content with time. The test results are listed in Table 2, Table 3 and Table 4, which show that the average mass of the ϕ50 mm × 100 mm specimen was 426.83 g after being stoved in an oven, the average free water content mass of the air drying ϕ50 mm × 100 mm specimen placed for 3 months was approximately 7.99 g, the average free water content mass of the ϕ50 mm × 100 mm specimen placed for 9 months was approximately 3.26 g, and the mass of the ϕ70 mm × 35 mm specimen placed for 9 months was 272.40 g after being stoved in an oven, the average free water content mass was approximately 2.10 g.

**Figure 2:**
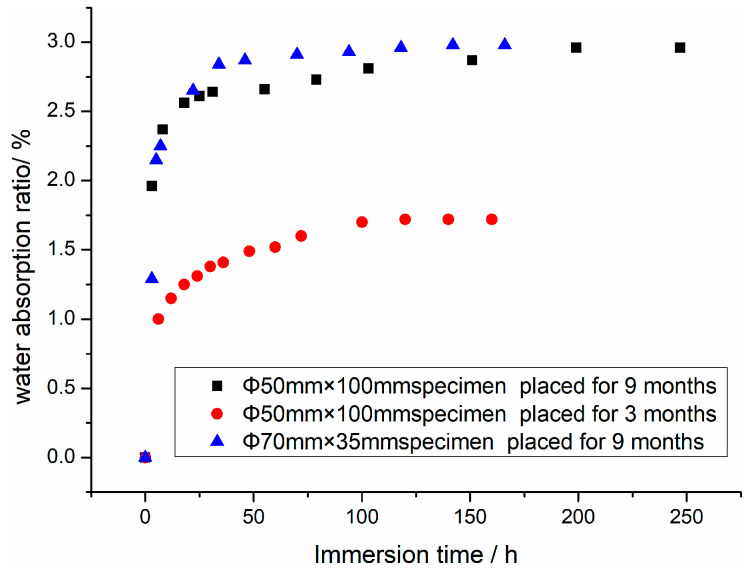
Variation of water absorption ratio with immersion time for specimens placed in a dry room for different times after immersion.

The variation pattern of mass and water absorption ratio with immersion time for three groups of specimens is shown in Figure 2, and the variation law of mass with immersion time for the ϕ50 mm × 100 mm specimen placed in a dry room at normal temperature for different times after immersion is shown in Figure 2.

**Figure 3:**
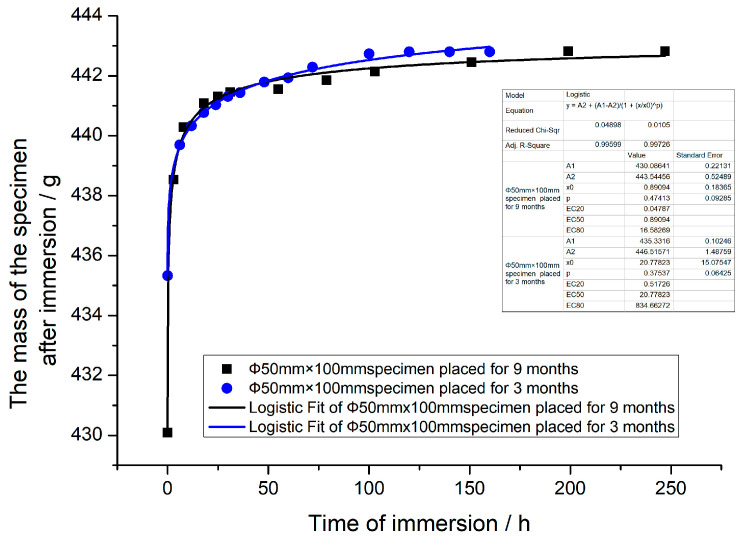
Variation of mass with immersion time for specimens of the same size placed in a dry room for different times after immersion.

From Table 2, Table 3 and Table 4 and Figure 2 and Figure 3, it can be found that for specimens of different sizes placed for the same time in a dry environment at the normal temperature, the initial and saturated water content ratios are the same, but the rate of change of water absorption mass is different. When specimens of the same sizes are placed in a dry environment at the normal temperature for different immersion times, although the initial mass of the dry specimens before immersion, rate of change of water absorption mass after immersion, and final water absorption mass at saturation are all different, the final mass of the two specimens at saturation is almost the same. That is to say, in spite of the difference in water absorption mass (or water absorption ratio) of specimens of the same size placed for different immersion times, the final water content mass (or water content ratio) of the two specimens at saturation were almost equal. The measured water content ratios at saturation for the three groups of specimens were basically the same, approximately 3.75%.

Therefore, the following conclusions can be drawn from the three sets of specimen-immersion experiments: the size and shape of the specimen affect the water absorption mass, rate of change of water absorption mass, water content ratio, and time to reach water saturation, but there is no effect on the water absorption and water content ratio at saturation. Although the time that the specimen was placed in a dry environment after the fabrication and maintenance affects the initial water content mass of the specimen, the water absorption mass after immersion, the water absorption ratio, the time to reach water saturation, and the rate of the change of the water absorption mass; it has no effect on the final water absorption mass, water content mass, and water content ratio of the specimen at saturation.

In addition, the differences in the aggregate type, mix ratio, and water reducing agent of concrete materials may affect its water absorption ratio and water content ratio. Therefore, to reduce the influence of the differences of the materials themselves on the research results, this study used the relative saturation ratio and relative water absorption ratio to describe the moisture level of concrete in a humid or water environment, and defined the relative saturation ratio S as the water content mass of the immersed specimen/the water content mass of the specimen at saturation. That is,

S = (M−M_d_)/(M_s_−M_d_) (1)

where M is the mass of the specimen after a predetermined time of immersion, M_d_ is the mass of the concrete specimen after being stoved in an oven, and M_s_ is the mass of the immersed concrete specimen. At the same time, the relative water absorption ratio W was used to describe the water absorption of concrete from the surrounding environment, which was defined as the real water absorption mass of the specimen/total water absorption mass of the specimen at saturation, i.e.,

W = (M−M_0_)/(M_s_−M_0_) (2)

where M_0_ is the mass of the air-drying concrete specimen before immersion.

## 3. Experimental Results

### 3.1. Quasi-Static Uniaxial Compression Tests on Concrete at High Temperature

**Figure 4:**
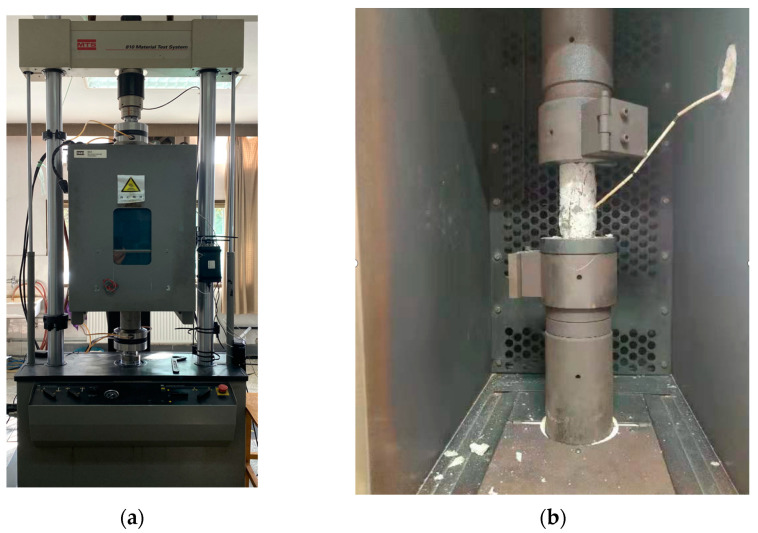
(**a**) MTS 810 test system’s installed heating chamber, (**b**) the interior of the heating chamber.

After production and curing, six groups of specimens (size ϕ50 mm × 100 mm) were placed in a dry environment for three months, which were then stoved in an oven, immersed for 2 h, 6 h, 60 h, immersed to full saturation, and without any treatment as the test subjects. The water content and water absorption of the immersed specimen used in the test are shown in Table 2. High temperature uniaxial quasi-static compression tests were carried out on the MTS 810 material test system and its supporting MTS 651 heating chamber produced by MTS Systems of the United States. And the loading rate was 10^−5^/s. The test system’s installed heating chamber is shown in Figure 4a and the interior of the heating chamber is shown in Figure 4b.

The size of the selected specimens was ϕ50 mm × 100 mm and the test temperatures were normal temperature (20 °C), 200 °C, 400 °C, and 520 °C, respectively. Before the test, we drilled holes in the core of concrete specimen column, and the depth of holes was about half of the length of the specimen. The thermocouple was inserted into the holes in the center of the specimens. At the set target temperatures (200 °C, 400 °C, 520 °C), 3–5 heating tests were carried out to determine the constant temperature time required to ensure the uniform distribution of the temperature inside the concrete after the heating chamber was heated to the target temperature. The results showed that the constant temperature time required to reach the target temperature inside the specimen was as follows: the constant temperature time is 60 min at 200 °C, 120 min at 400 °C, and 150 min at 520 °C. In addition, before the experiment, it was necessary to check the insulation of the heating chamber, and insulation cotton was used to wrap the heating chamber, so as to ensure the heating efficiency and the accuracy of the test results.

**Figure 5:**
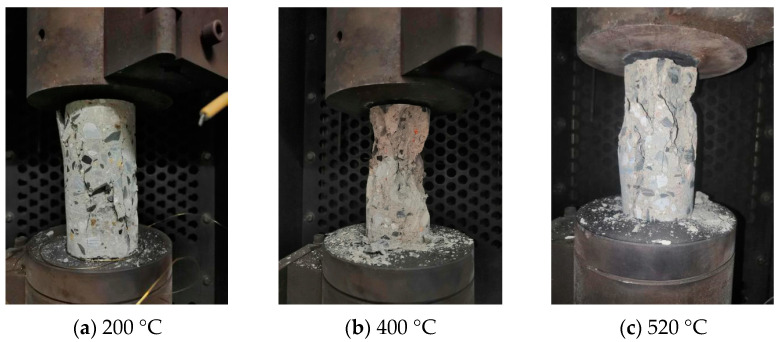
Typical damage morphology of concrete specimens immersed for 60 h at different temperatures.

The quasi-static uniaxial compressive strengths of the concrete specimens with different water contents at different temperatures were obtained, and the specimens had typical damage morphology after the tests, as shown in Figure 5.

During the heating process of the specimen, the color of the specimen changed with the rise in temperature. The color of the specimen did not change much before the temperature reached 200 °C, but when the temperature reached 400 °C and 520 °C, the specimen presented a light yellow and gray-white color, respectively. After the specimen reached the predetermined temperature, there were no obvious cracks before loading. However, in some reports [34] of high-temperature experiments of concrete, high-temperature burst occurred before the temperature reached 500 °C. This may be due to the fast heating rate and large volume of the specimen, which lead to uneven temperature distribution and large temperature gradient inside the specimen. The thermal stress exceeds the tensile strength of the concrete itself. In this case, the specimen may undergo high-temperature burst. In addition, the volume of fragmentation after specimen destruction decreased with the increase in experimental temperature.

### 3.2. Temperature Softening Effect of Compressive Strength of Dry Concrete

The compressive strength of concrete materials shows a significant softening effect at high temperatures, and the effect of temperature on the strength of concrete materials is generally characterized by the variation pattern of softening factor, K_T_, with temperature. The temperature softening factor, K_T_, of compressive strength is often defined as the ratio of the high temperature quasi-static uniaxial compressive strength, σ_T_, of air-drying concrete to the normal temperature quasi-static uniaxial compressive strength, σ_0_, i.e., K_T_ = σ_T_/σ_0_.

**Figure 6:**
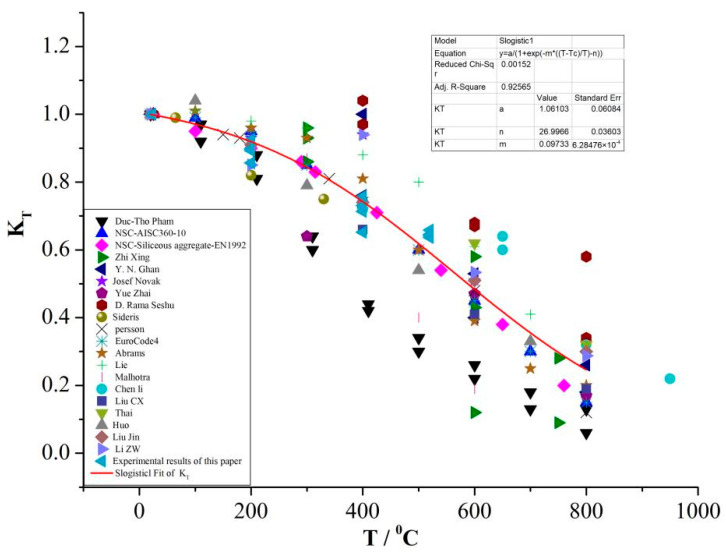
Temperature softening effect of compressive strength of air-drying concrete.

In this study, the experimental data of the temperature softening factor, K_T_, with temperature for air-drying concrete specimens were obtained from the above experiments, as shown in Figure 6. The experimental data are basically within the discrete range of the existing experimental results [10,11,12,13,14,15,16,17,18,19,20,21,22,23,24,25,26,27,28,29], and are in good agreement with the K_T_–T curves obtained by Ping Li [30] by summarizing and analyzing the uniaxial quasi-static compression experimental data of concrete materials at different temperatures in recent decades. It can be observed that the compressive strength of concrete materials shows a nonlinear monotonic decrease with the rise in temperature.

The relationship between the temperature softening factor, K_T_, and the temperature, T, obtained is given by Ping Li [30]:

(3) $$K_{T}=\frac{a}{1+e^{-m(\frac{T-T_{c}}{T_{c}}-n)}},$$

where T_c_ is the reference temperature, T_c_ = 20 °C, a = 1.1, m = 0.1, and n = 27.

### 3.3. Water Softening Effect on the Compressive Strength of Concrete at Normal Temperature

**Figure 7:**
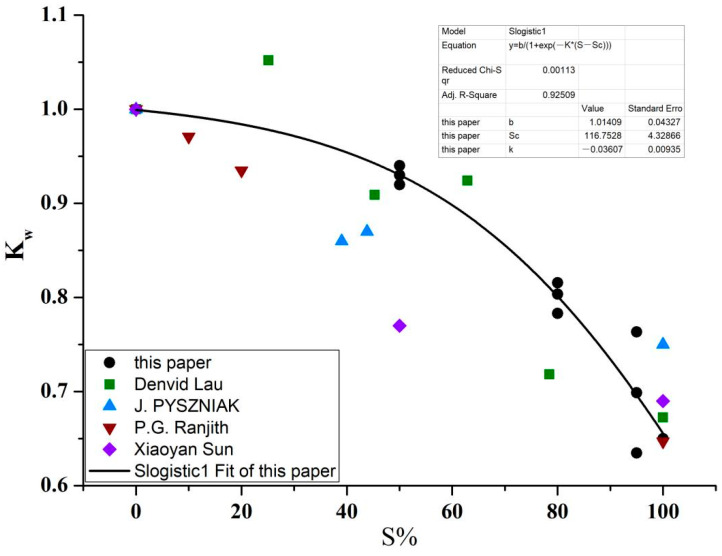
The relation between the water softening factor, K_w_, and the relative saturation ratio, S.

The quasi-static uniaxial compressive strengths of concrete specimens with different water contents (expressed by relative saturation ratio in this paper) at normal temperature were obtained, along with the variation curves of the water softening factor, K_w_ (K_w_ = $\sigma_{w}/\sigma_{d}$, where *σ_w_* is the compressive strength of the immersed specimens and σ_d_ is the compressive strength of the specimens stoved in an oven) with the relative saturation ratio S. The comparison between the experimental results in this paper and those in existing literature [35,36,37,38] is shown in Figure 7. As can be observed, the compressive strength of the concrete material at normal temperature shows an obvious non-linear decreasing trend with the increase in water content, and the fitting equation of the relationship between the variation factor, K_w_, and the relative saturation ratio, S, is:

(4) $$K_{w}=\frac{b}{1+e^{-k(S-S_{c})}},$$

where parameters b = 1.0, S_c_ = 116.8, and k = −0.04.

### 3.4. Changes in the Compressive Strength of Concrete under Combined Heat–Moisture Conditions

The above high temperature compression experiments on dry concrete specimens and normal temperature compression experiments on immersed concrete specimens verify the temperature softening effect and water softening effect on the compressive strength of concrete, respectively. However, under combined heat and moisture conditions, the water inside the concrete will undergo phase transformation and disappear, and the variation pattern of the compressive strength of the concrete material is yet to be determined. In this study, on the basis of the high temperature uniaxial quasi-static compression experiments of dry and wet concrete, the variation factor of the compressive strength of the concrete material under combined heat and moisture conditions is defined as DIF = σ_Ts_/σ_d_, where σ_Ts_ is the compressive strength of concrete materials with different water contents (or different relative saturation ratios, S) at temperature, T, and σ_d_ is the compressive strength of concrete at normal temperature after being stoved in an oven. The variation relationship between the heat–moisture combined effect factor DIF and the relative saturation ratio is obtained and shown in Figure 7.

**Figure 8:**
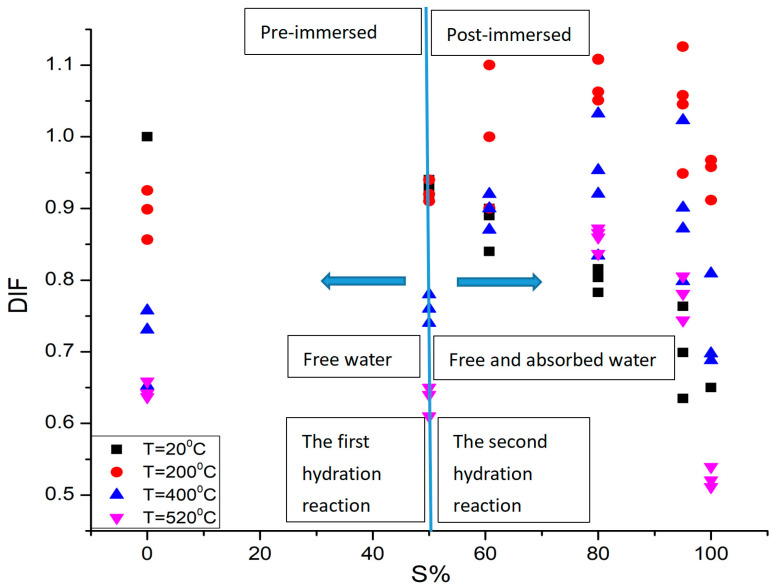
Relationship between the heat–moisture combined effect factor DIF and the relative saturation ratio S of the specimens for the compressive strength of the immersed concrete specimens.

We found two interesting phenomena from Figure 8: (1) The variation pattern of the compressive strength of the immersed concrete is significantly different at high temperature compared with that at normal temperature. The compressive strength of concrete at normal temperature decreases monotonically with the increase in water content, while the compressive strength of concrete at high temperature shows a parabolic variation trend from rise to decline with the increase in water content; (2) the influence of water content on the variation factor of compressive strength is significantly different before and after immersion. The compressive strength of the specimens stoved in an oven (S = 0%) decreases monotonically with the rise in temperature, whereas the high temperature compressive strength of the air-drying specimens placed in the dry environment for three months (S is approximately 50%) is slightly higher than that of the specimens stoved in an oven. The effect of free water content in the air-drying specimens on the variation factor DIF of the concrete compressive strength at high temperature is not significant. However, for the specimens after immersion, the effect of water content on DIF is more significant, that is, there is an obvious difference between the effect of the free water stored during the first hydration process and the water absorbed during the second hydration process on the compressive strength of concrete at high temperature. This also shows that the effect of temperature and water on the compressive strength of concrete under combined heat and moisture conditions are coupled with each other. That is, the DIF is the heat and moisture coupling effect factor of the compressive strength of concrete.

Despite the fact that the free water content stored inside the concrete differs when it is placed in the dry normal temperature environment for different times after production and curing, and the free water content decreases with the increase in placing time, the effect of the free water retained in the concrete during the first hydration process on the compressive strength at high temperature is not significant compared to that of the water absorbed during the second hydration process. Therefore, this study mainly addressed the variation pattern of the compressive strength of the immersed concrete specimens at high temperature and found the coupling relationship among water absorption ratio, temperature, and compressive strength of concrete specimens undergoing the second hydration. This has a more practical significance not only for the prediction of compressive strength of concrete when working in a hot and humid environment but also for the design of structural protection.

**Figure 9:**
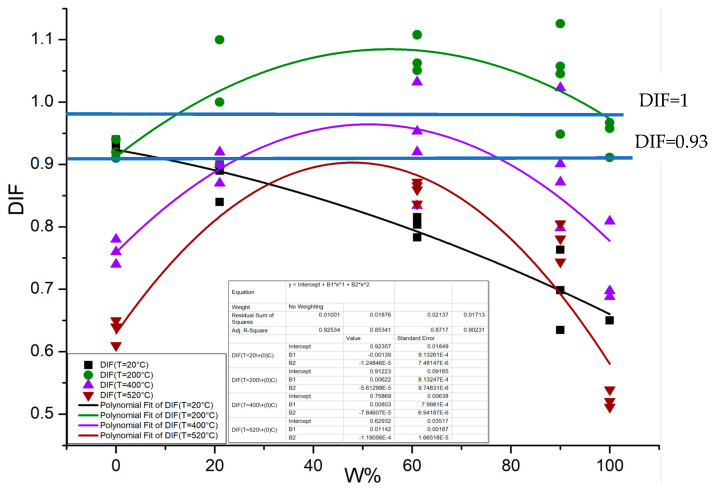
Curve of the heat–moisture coupling effect factor DIF of concrete compression strength versus relative water absorption ratio W_ab_.

The relationship curves between the relative water absorption ratio W_ab_ and the heat–moisture coupling effect factor DIF of the compressive strength of concrete specimens at different temperatures are obtained by fitting the above experimental results, as shown in Figure 9, where the specimens were stoved in an oven have DIF = 1 and the air-drying specimens have DIF = 0.93 at normal temperature.

The following patterns can be observed from Figure 9.

(1) The variation patterns of the compressive strength of immersed concrete at high temperature and normal temperature are remarkably different. At high temperature, the quasi-static compressive strength of immersed concrete shows a trend from rise to decline with the increase in relative water absorption ratio of the specimen, which is from approximately 45% to 65%. The high temperature compressive strength of concrete reaches a peak, and after that the strength decreases rapidly. Further, as the water absorption increases, the higher the temperature of concrete is, the faster the rate of strength decrease is after the peak. The relationship among the heat–moisture coupling effect factor DIF, the corresponding relative water absorption ratio, and temperature at the peak can be obtained by the fitted curve. As can be observed in Table 5, when T ≥ 200 °C, the peak of DIF decreases with the increase in temperature, and the corresponding relative water absorption ratio, $W_{ab}^{c}$, also decreases as the DIF at each temperature reaches the peak.
(2) Compared with the compressive strength of air-drying concrete at normal temperature, the high-temperature compressive strength of immersed concrete increases in a certain range with the increase in water absorption mass (or relative water absorption), but the increase in the value of the compressive strength and the range of the relative water absorption ratio corresponding to the increase in compressive strength are temperature dependent. When T = 200 °C, the high-temperature compressive strength of the air-drying concrete specimens is slightly lower than their normal-temperature compressive strength, and the high-temperature compressive strength of the immersed concrete specimens is higher than the normal-temperature compressive strength of the air-drying concrete specimens. Even in a larger range of relative water absorption ratio (approximately 10% to 90%), their compressive strength is higher than that of the concrete stoved in an oven at normal temperature. When T = 400 °C, the compressive strength of the immersed concrete is lower than that of the concrete stoved in an oven at normal temperature, and it is only slightly higher than the compressive strength of the air-drying concrete specimens at normal temperature near the peak. Further, the corresponding range of the relative water absorption ratio is significantly narrower than that at T = 200 °C. When T = 520 °C, the compressive strength of the immersed concrete is lower than that of the air-drying concrete specimens at normal temperature.
(3) Under the same relative water absorption ratio, the compressive strength of immersed concrete at T = 200 °C is higher than that at normal temperature (it is only slightly lower than the compressive strength at normal temperature when W_ab_ is less than 2%, approximately). At T = 400 °C, when the relative water absorption ratio, W_ab_, is less than 22%, the compressive strength is higher than that at normal temperature and with the same relative water absorption ratio. At T = 520 °C, when the relative water absorption ratio, W_ab_, is greater than 33% and less than 90%, the compressive strength is higher than that at normal temperature and with the same relative water absorption ratio. However, when W_ab_ is greater than 90%, the compressive strength is lower than that at normal temperature with the same water absorption ratio.

Before the quasi-static high-temperature compressive strength of concrete reaches its peak, the intersection point of the DIF (T = 200 °C, T = 400 °C, T = 520 °C)−W_ab_ curve and the DIF (20 °C)−W_ab_ curve shifts back with the increase in temperature. The DIF (20 °C)−W_ab_ curve reflects the water softening effect of the compressive strength of concrete under water immersion conditions at normal temperature. At this intersection, the heat–moisture coupling effect at temperature T is equivalent to the water absorption softening effect at normal temperature. Beyond this intersection point, within a certain range of relative water absorption ratio, the temperature plays a role in reducing the water softening effect, which is macroscopically manifested as the temperature strengthening phenomenon of the immersed concrete. This occurs because the free water absorbed during the material immersion process undergoes phase change and is lost at high temperature, thus weakening the softening effect of water on the compressive strength and leading to the result that the high-temperature compressive strength of immersed concrete is higher than that at normal temperature. This “pseudo-temperature strengthening effect” is a distinctive feature that distinguishes the thermodynamic response of immersed concrete from that of dry concrete (including air drying concrete and concrete stoved in an oven).

**Figure 10:**
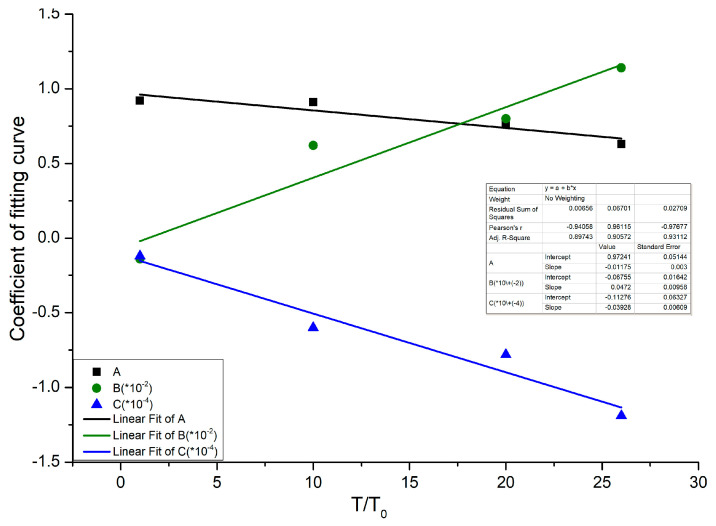
The variation trend of fitting coefficient with temperature.

(4) The fitted equation of the heat–moisture coupling effect factor DIF and relative water absorption ratio, W_ab_, is:

DIF(T) = A(T) + B(T)W_ab_ + C(T)W_ab_^2^, (5)

where the fitted parameters A(T), B(T), and C(T) are only functions of temperature, whose values taken at each temperature are listed in Table 6. Their changing trends with temperature are shown in Figure 10.

The variation relationship of A(T), B(T), and C(T) with respect to temperature is obtained from the experiment, as follows:

A(T) = 0.97 − 0.012 (T/T_0_), (6)

B(T) = −6.8 × 10^−4^ + 4.7 × 10^−4^ (T/T_0_), (7)

C(T) = −1.1 × 10^−5^ − 3.9 × 10^−6^ (T/T_0_), (8)

where T_0_ = 20 °C.

The equation reflects the change pattern of compressive strength of the concrete material under the influence of the heat–moisture coupling effect. When DIF(T) = 1, it means that, under the action of the heat–moisture coupling effect, the compressive strength of this concrete specimen and that of the concrete specimen stoved in an oven at normal temperature are equal. In this case, the heat–moisture coupling effect has the same effect on the crack growth and healing inside the material. Macroscopically, the heat–moisture coupling effect has no influence on the compressive strength of concrete at this temperature and moisture condition. When DIF(T) > 1, it indicates that the high-temperature compressive strength of immersed concrete under the action of the heat–moisture coupling effect is greater than that of the concrete stoved in an oven at normal temperature. In this case, the role of the heat–moisture coupling effect in promoting the healing of cracks within the material is greater than the role in promoting the growth of cracks, which is macroscopically manifested as the increase in the compressive strength of concrete at this temperature and moisture condition. When DIF(T) < 1, the compressive strength of concrete under the action of the heat–moisture coupling effect is less than that of the concrete stoved in an oven at normal temperature. In this case, the role of the heat–moisture coupling effect in promoting the growth of cracks within the material is greater than the role in promoting the healing of cracks, which is macroscopically manifested as the attenuation of the compressive strength of concrete at this temperature and moisture condition.

**Figure 11:**
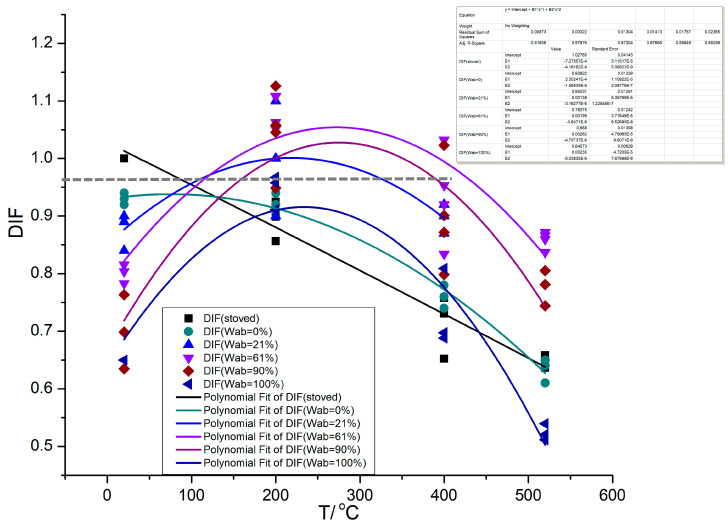
Variation pattern of the heat–moisture coupling effect factor DIF of compression strength with temperature for concrete specimens with different relative water absorption ratio W_ab_ and concrete specimens stoved in an oven.

The variation pattern of the heat–moisture coupling effect factor DIF with temperature for the concrete specimens with different relative water absorption ratio and concrete specimens stoved in an oven are shown in Figure 11.

From the variation curve of DIF−T in Figure 11, the following is found:

(1) The heat–moisture coupling effect factor DIF of quasi-static compressive strength of concrete stoved in an oven decreases linearly with increasing temperature, T. The fitted equation for the DIF–T relationship is

DIF (stoved) = 1.02 − 0.02T*,(9)

where T* = T/T_0_ and T_0_ is the reference temperature (T_0_ = 20 °C). DIF (stoved) = σ_T_/σ_d_, where σ_d_ is the quasi-static normal temperature compressive strength of the concrete specimen stoved in an oven and σ_T_ is the quasi-static high temperature compressive strength of the concrete specimen stoved in an oven. Equation (9) reflects the temperature softening effect of concrete specimens that are not affected by free water.

(2) From the DIF (W_ab_ = 0%)−T change curve of the heat–moisture coupling effect factor DIF (W_ab_ = 0%) of the quasi-static compressive strength of the air-drying concrete (W_ab_ = 0%) with temperature, T, it can be observed that, compared to the concrete specimens stoved in an oven, the DIF (W_ab_ = 0%)−T curve shows an obvious non-linear decline characteristic, and its decline rate is more gentle until 350 °C. Some researchers [14,17] found that the compressive strength of the concrete specimen even increases between 200 and 400 °C, and the main reason is that the air drying of concrete specimens in the experiment is not sufficient, and the free water stored inside the specimen is excessively high. The decline gradually increases after 350 °C, especially after 400 °C, where the rate of decline exceeds that of the concrete specimens stoved in an oven.
(3) The heat–moisture coupling effect factor DIF of the compressive strength of immersed concrete shows a trend from rise to decline with the increase in temperature. The DIF reaches its maximum value between 200 and 300 °C, and then decreases rapidly, and the rate of decrease is faster than that of concrete air drying or that stoved in an oven. The peak of DIF declines with the increase in water absorption, which also indicates that the moisture effect on material strength is not fully reversible. Based on the fitting curves, it is found that when the DIF reaches its peak, the corresponding peak temperatures of the concrete specimens with the relative water absorption ratio of W_ab_ = 60% and W_ab_ = 90% are close to each other, whereas the peak of the saturated concrete specimen (W_ab_ = 100%) is significantly lower than that of the specimens with the above two water absorption ratios and the temperature corresponding to the peak is smaller.

In addition, after the heat–moisture coupling effect factor DIF of the immersed concrete reaches its peak, the decline rate of DIF accelerates with the increase in water absorption of the specimens Therefore, the specimens with greater water absorption are more likely to burst at high-temperature.

(4) From the fitted experimental curves, it can be observed that the strength of concrete with the relative water absorption ratios of 60% and 90% is greater than that of stoved concrete at normal temperature within a certain temperature interval corresponding to the vicinity of the DIF peak. For concrete specimens with 60% relative water absorption ratio, when the temperatures range from approximately 150 to 400 °C, their compressive strengths are greater than that of stoved concrete specimens at normal temperature, that is, DIF > 1; when the temperature is between 100 and 450 °C, their compressive strengths are greater than that of air-drying concrete specimens at normal temperature; when the temperature is between 80 and 520 °C, their compressive strengths are greater than that of air-drying concrete specimens at the same high temperature. For concrete specimens with 90% relative water absorption ratio, when the temperatures range from approximately 200 to 350 °C, their compressive strengths are greater than that of stoved concrete specimens at normal temperature, that is, DIF > 1; when the temperature is between 140 and 420 °C, their compressive strengths are greater than that of air drying concrete specimens at normal temperature; when the temperature is between 135 and 520 °C, their compressive strengths are greater than that of air-drying concrete specimens at the same high temperature. The high temperature compressive strength of fully saturated concrete specimens is lower than the normal temperature compression strength of both air-drying specimens and specimens stoved in an oven. The compressive strength of fully saturated concrete specimens is greater than that of air-drying concrete at the same temperature ranging from 200 to 400 °C.

## 4. Mechanism Analysis

**Figure 12:**
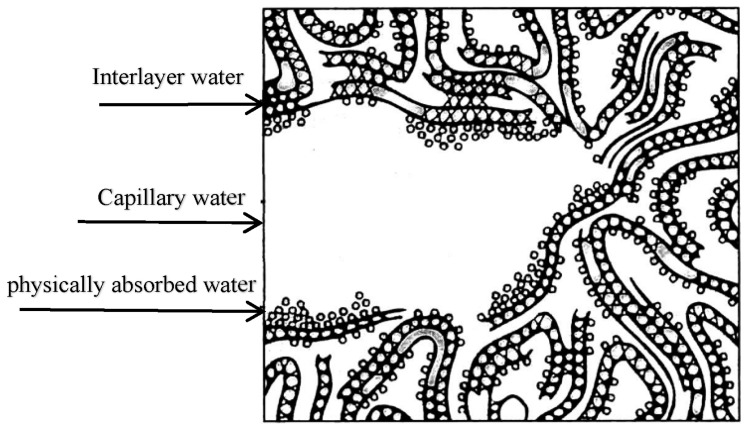
Water type in concrete materials.

After water absorption in concrete, the water inside the specimen undergoes a series of physical and chemical processes during heating: water absorbed during immersion is evaporated, then free water stored in the specimen before immersion is evaporated → physically adsorbed water adsorbed on the surface of the solid phase is lost due to drying → water between the layers of the C-S-H structure is lost due to intense drying → chemically bound water is lost along with hydride decomposition. According to the Feldman–Sereda [39] model, the water type in the concrete material is shown in Figure 12.

At normal temperature, approximately 50% of the hydrogen bonds of water are broken, and in this state, the surface charge of the material is unsaturated. Thus, the surface energy increases, which leads to surface tension and further causes a large number of molecules that tend to agglomerate together. The water that accumulates at the crack tip has a wedge-like splitting effect on crack extension. After curing, the concrete will shrink and form cracks, G0 (including reversible cracks G1 and irreversible cracks G2), due to water loss in its interior under normal temperature and air-drying conditions, especially in the interface transition zone between the aggregate and cement mortar, where more microcracks and microporosity exist. Because of this, the interface transition zone is the weakest link in the strength chain and is usually considered as the strength limiting phase in concrete, which plays a key role in the compressive strength of concrete. Assuming that the evolution process of reversible and irreversible cracks within the material is independent. After dry concrete is immersed in water, a small number of reversible cracks, G1, are closed, and a large number of irreversible cracks, G2, are continuously derived, grown, and connected on the basis of the original damage nucleus caused by the wedge splitting effect of water, Finally, a new crack group, G, with a larger scale and number will be formed. Therefore, the compressive strength of immersed concrete specimens is less than that of dry concrete at normal temperature.

**Figure 13:**
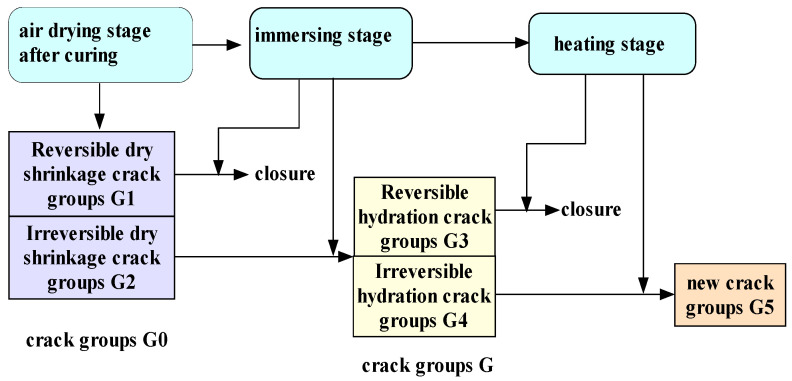
Schematic of crack evolution.

The new cracks group, G, after water immersion also contain reversible crack part G3 and irreversible crack part G4. As the temperature rises, the water inside the material will undergo a series of physical and chemical reactions. The volume expansion of water from liquid phase to gas phase at high temperature may lead to the increase in pore tension in the cracks. However, because of the existence of a large number of microcracks, G, inside the material during the hydration process after water immersion, the gas generated by the water phase transition is rapidly transferred to the surrounding medium through these microcracks. Moreover, this process of mass and heat transfer makes the temperature distribution inside the immersed specimen more uniform than that of the dry specimen, and the thermal stress caused by the temperature gradient is smaller. As indicated by the results of the present experiments in the temperature range of 20 to 520 °C, the pore tension inside the immersed concrete specimen is not large, which has also been proved by the test results of pore pressure of concrete materials under the combination of heat and moisture conducted by Mindeguia [33]. In addition, the reversible cracks, G3, formed during the second hydration after immersion close with the loss of moisture inside the material at high temperatures. Meanwhile, the irreversible cracks, G4, continue to expand with the increase in temperature and the occurrence of physical and chemical reactions inside the material to form new crack groups, G5. The above crack evolution process can be divided into three stages as follows, which are schematically shown in Figure 13.

(1) Air-drying stage at normal temperature after production and curing

At this stage, the dry shrinkage phenomenon occurs in the specimen due to air drying and water loss, forming an initial crack group, G0, which includes reversible dry shrinkage cracks, G1, and irreversible dry shrinkage cracks, G2.

(2) Immersion stage

During water immersion of the specimen, the reversible dry shrinkage cracks, G1, heal while the irreversible dry shrinkage cracks, G2, expand and connect under the wedge splitting action of water, forming a larger size crack cluster, G, which includes reversible hydration cracks, G3, and irreversible hydration cracks, G4.

(3) Heating phase

At high temperature, the free water inside the material is gradually lost, the reversible hydration cracks, G3, close, while the irreversible hydration cracks, G4, further expand with a series of physicochemical reactions inside the material to form a new crack group, G5.

At normal temperature, the amount of cracks formed by dry shrinkage of the specimen is smaller than the amount of hydration cracks formed by water absorption of the specimen, i.e., G0 < G, and thus the compressive strength of immersed concrete is smaller than that of dry concrete. However, at high temperature, the crack evolution process is influenced by the coupling effect of temperature and moisture. With the increase in temperature, the reversible hydration cracks, G3, in concrete specimens with different water contents began to heal, and the compressive strength of wet concrete gradually rises. In the process of reversible hydration crack healing, the amount of cracks (G3 + G4) gradually decreases. The compressive strength of wet concrete with the same water content at high temperature is greater than that at normal temperature. When the amount of cracks (G3 + G4) < G0, the high temperature compression strength of wet concrete is greater than the compression strength of dry concrete at normal temperature. When G3 is completely healed, the high temperature compression strength of wet concrete reaches its peak, and the irreversible hydration crack, G4, began to develop rapidly, forming a new crack group, G5. When G5 < G0, the wet concrete high temperature compression strength is greater than the compression strength of dry concrete at normal temperature. When G5 = G0, the wet concrete high temperature compression strength is equal to the compression strength of dry concrete at normal temperature. When G5 > G0, the wet concrete high temperature compression strength is lower than the compression strength of dry concrete at normal temperature. From the DIF−T change curve, it can be found that after the compressive strength of wet concrete reaches its peak, DIF decreases with the increase in temperature, The higher the water content is, the faster the rate of DIF decline, indicating that the expansion rate of crack group G5 is significantly greater than the crack expansion rate of the dry concrete material at the same temperature. Further, the higher the relative saturation rate of concrete after water absorption, the higher the propagation rate of the G5 crack group after the compression strength reaches the peak, and the more likely the high temperature bursting phenomenon in specimens is to occur.

## 5. Conclusions

In this study, by carrying out experimental studies on quasi-static uniaxial compression of concrete materials at different temperatures and different water contents, the coupling relationship among compression strength of concrete, temperature, and water absorption ratio is determined, and the mechanism of the heat–moisture coupling effect on the compression strength of concrete is analyzed based on the experimental results. The following conclusions are obtained.

(1) Under combined heat and moisture conditions, the change in concrete compressive strength no longer shows a monotonic decay law, but a parabolic variation characterized by an increase followed by a decrease. When the relative water absorption ratio of the specimen is approximately 45% to 65% and the temperature is approximately 200 to 300 °C, compressive strength of the immersed concrete reaches its peak.
(2) There is a significant difference in the effect of water on the compressive strength of concrete before and after immersing. That is, the free water stored during the process of production, curing, and air drying (i.e., first hydration) and the water absorbed during the process of immersing (i.e., second hydration) have different effects on the crack evolution of concrete materials, and the effect of water absorption of concrete during the second hydration is more significant on its compression strength. However, the microscopic mechanism of the effect of the two hydration processes on the evolution of microcracks needs to be further investigated in depth.
(3) The expansion and closure of cracks within the concrete material are the result of the heat and moisture coupling effect. The compression strength of the concrete rises when the crack closure rate dominates, and conversely, the compression strength of the concrete material decreases when the crack expansion rate dominates. On the basis of previous studies and combined with our experimental results, we have given a reasonable explanation for the evolution process of microcracks under the combined heat and moisture conditions. However, more micro-scale experimental evidence is needed. This is also the work we are currently carrying out, and some results have been obtained, which we believe will be published in the near future. In addition, the specimen size used in the heat and moisture coupling experiment and theoretical research of concrete materials in this paper is slightly small. In the heat and mass transfer process, the internal crack evolution law caused by the temperature and moisture gradients of large-size concrete specimens under combined heat and moisture conditions may differ from that of small specimens. These are more prone to high-temperature cracking due to the more significant temperature and moisture gradients under the heat and moisture coupling conditions. The change pattern of the heat–moisture coupling effect factor of the large-size concrete specimens have to be further verified.
(4) The equation for the heat–moisture coupling effect factor DIF of the compressive strength, temperature, T, and relative water absorption ratio, W_ab_, is:

DIF = A(T) + B(T)W_ab_ + C(T)W_ab_^2^

where the parameters A(T), B(T), and C(T) are only functions of temperature, and the equation reflects the coupling effect of heat and moisture on the compressive strength of concrete. The results of this study can provide theoretical guidance and scientific reference for the design of concrete protective structures working in high temperature and high moisture environments.

## Figures and Tables

**Table 1:** Mix proportions of concrete specimen.

Cement (kg/m^3^)	Sand (kg/m^3^)	Aggregate (kg/m^3^)	Water (kg/m^3^)	Superplasticizer (kg/m^3^)
425	600	1132	184	8

**Table 2:** Water absorption of the ϕ50 mm × 100 mm concrete specimens immersed for different times and placed in a dry room at normal temperature for 3 months.

Immersion Time/h	Specimen Mass/g	Water Absorption Mass/g	Water Absorption Ratio/%	^1^ Relative Water Absorption Ratio/%	Water Content Mass/g	Water Content Ratio/%	^2^ Relative Saturation Ratio/%
0	434.82	0	0	0	7.99	1.87	50.03
2	436.53	1.71	0.39	21.43	9.7	2.27	60.74
6	439.69	4.87	1.12	61.03	12.86	3.01	80.53
12	440.33	5.51	1.28	69.05	13.5	3.16	84.53
18	440.77	5.95	1.38	74.56	13.94	3.27	87.29
24	441.03	6.21	1.43	77.82	14.2	3.33	88.92
30	441.31	6.49	1.49	81.33	14.48	3.39	90.67
36	441.44	6.62	1.52	82.96	14.61	3.42	91.48
48	441.79	6.97	1.60	87.34	14.96	3.50	93.68
60	441.93	7.11	1.64	89.10	15.10	3.54	94.55
72	442.29	7.47	1.72	93.61	15.46	3.62	96.81
100	442.73	7.91	1.82	99.12	15.90	3.73	99.56
120	442.80	7.98	1.84	100	15.97	3.74	100
140	442.80	7.98	1.84	100	15.97	3.74	100
160	442.80	7.98	1.84	100	15.97	3.74	100

Note: ^1^ Relative water absorption ratio = (water absorption mass of specimen/total water absorption mass of specimen at saturation) × 100%. ^2^ Relative saturation ratio = (water mass of specimen/water mass of specimen at saturation) × 100%.

**Table 3:** Water absorption of the ϕ50 mm × 100 mm concrete specimens immersed for different times and placed in a dry room at normal temperature for 9 months.

Immersion Time/h	Specimen Mass/g	Water Absorption Mass/g	Water Absorption Ratio/%	^1^ Relative Water Absorption Ratio/%	Water Content Mass/g	Water Content Ratio/%	^2^ Relative Saturation Ratio/%
0	430.09	0	0	0	3.26	0.76	20.39
1	433.23	3.14	0.73	24.67	6.4	1.50	40.03
3	438.53	8.44	1.96	66.30	11.7	2.74	73.17
8	440.29	10.2	2.37	80.13	13.46	3.15	84.18
18	441.09	11	2.56	86.41	14.26	3.34	89.18
25	441.31	11.22	2.61	88.14	14.48	3.39	90.56
31	441.46	11.37	2.64	89.32	14.63	3.43	91.49
55	441.55	11.46	2.66	90.02	14.72	3.45	92.058
79	441.85	11.76	2.73	92.38	15.02	3.52	93.93
103	442.14	12.05	2.81	94.66	15.31	3.59	95.75
151	442.45	12.36	2.87	97.09	15.62	3.66	97.69
199	442.82	12.73	2.96	100	15.99	3.75	100
247	442.82	12.73	2.96	100	15.99	3.75	100

Note: ^1^ Relative water absorption ratio = (water absorption mass of specimen/total water absorption mass of specimen at saturation) × 100%. ^2^ Relative saturation ratio = (water mass of specimen/water mass of specimen at saturation) × 100%.

**Table 4:** Water absorption of the ϕ70 mm × 35 mm concrete specimens immersed for different times and placed in a dry room at normal temperature for 9 months.

Immersion Time/h	Specimen Mass/g	Water Absorption Mass/g	Water Absorption Ratio/%	^1^ Relative Water Absorption Ratio/%	Water Content Mass/g	Water Content Ratio/%	^2^ Relative Saturation Ratio/%
0	274.52	0	0	0	2.10	0.76	20.58
3	278.06	3.54	1.29	43.28	5.64	2.06	54.95
5	280.42	5.90	2.15	72.13	8.00	2.93	77.86
7	280.69	6.17	2.25	75.43	8.27	3.03	80.49
22	281.78	7.26	2.65	88.75	9.36	3.43	91.07
34	282.31	7.79	2.84	95.23	9.89	3.62	96.21
46	282.39	7.87	2.87	96.21	9.97	3.65	96.99
70	282.51	7.99	2.91	97.68	10.09	3.70	98.16
94	282.57	8.05	2.93	98.41	10.15	3.72	98.74
118	282.63	8.11	2.96	99.14	10.21	3.74	99.32
142	282.70	8.18	2.98	100	10.28	3.77	100
166	282.70	8.18	2.98	100	10.28	3.77	100

Note: ^1^ Relative water absorption ratio = (water absorption mass of specimen/total water absorption mass of specimen at saturation) × 100%. ^2^ Relative saturation ratio = (water mass of specimen/water mass of specimen at saturation) × 100%.

**Table 5:** The relationship among peak of DIF, temperature T, and the corresponding relative water absorption ratio, $W_{\mathrm{ab}}^{c}$, at the peak of DIF.

Peak of DIF	Temperature T/°C	$W_{\mathrm{ab}}^{c}$
1.10	200	58%
0.96	400	52%
0.90	520	47%

**Table 6:** Fitted parameter values.

T/°C	A	B (×10^2^)	C (×10^4^)
20	0.92	−0.14	−0.12
200	0.91	0.62	−0.6
400	0.76	0.80	−0.78
520	0.63	1.14	−1.19

## Data Availability

The authors confirm that the data supporting the findings of this study are available within the article.
